# Supplementary material for: dicast: a machine learning method for accurate structural variant detection from short-read sequencing data
Source: Genome Biol. 2026 Sep 16;27:292. doi: 10.1186/s13059-026-04280-y (PMC13584385; doi:10.1186/s13059-026-04280-y)
Supplement: Supplementary file 1 [file 13059_2026_4280_MOESM1_ESM.docx]

# Supplementary Figures

**Fig. S1: Problem Definition and Workflow Overview.** A) Illustration of different types of structural variants. The top arrow represents the reference genome, the bottom arrow represents the patient genome. B) Limitations in structural variant detection. Pairwise overlap between sequencing technologies and Illumina short-read callers on sample S1. Each bar is the Jaccard index of the two sets of SV calls, i.e., the fraction of calls shared by both methods out of those called by either. Low values indicate that most calls are private to a single technology or caller. *IGV* screenshots depict two example variants in HG002 which show clear alignment patterns corresponding to true heterozygous deletions but which are not part of the HGSVC Freeze 4.0 benchmark set. C) Overview of the performed steps in the paper.

**Fig. S2: Composition of the ground truth SV dataset.** A) Number of confirmed variants per number of supporting technologies. Each dot represents one sample in the benchmark dataset. The pie charts beneath each number of supporting technologies show the distribution of variants detected by each technology. B) Number of conflicting variants per number of supporting technologies. C) Fraction of ground truth variants overlapping with genomic annotations, stratified by SV type.. D) Fraction of ground truth variants per confirmation method. Used strategies to confirm a variant were: Two technology support, two long-read method support, present in public database, manual curation.

**Fig. S3: Per-technology support and size distribution of the ground truth.** A) Fraction of per sample ground truth variants supported by each technology stratified by SV type. B) Per-sample size distribution of ground-truth variants stratified by SV type and binned into 51–100 bp, 101 bp–1 kb, 1–10 kb, and >10 kb.

**Fig. S4 Comparative analysis of methods and sequencing technologies for structural variant detection.** A) Precision and recall for duplication detection. The blue line represents precision, while the orange line represents recall. The confidence intervals and error bars indicates the standard deviation for precision and recall across n=9 samples for SRS, LRS, OGM; n=5 for LiRS I; n=6 for LiRS II. The horizontal bar plots compare the precision and recall when combining multiple methods within one technology. Striped bars describe a more than 2/n caller strategy, while non-striped bars describe a union strategy. (Short-Read: Illumina, Long-Read: PacBio CLR, Linked-Read I: 10x Genomics, Linked-Read II: TELL-Seq, Optical Mapping: Bionano) B) Precision and recall for inversion detection. C) Evaluation of combining multiple sequencing technologies for SV detection. Multiple methods within one technology were combined using the union strategy. Error bars indicate standard deviation across all respective samples. D) Precision (blue) and recall (orange) across technologies, SV types, and size categories. Multiple methods within one technology were combined using the union strategy. Shaded areas reflect the standard deviation across all respective samples.

**Fig. S5 Comparative analysis of methods and sequencing technologies for structural variant detection.** A) Log fold change of recall (top) and precision (bottom) per sequencing technology relative to the technology's overall baseline (dashed line at 0), across 11 genomic annotations: CpG islands, LINEs, LTRs, low-complexity regions, retroposons, SINEs, STRs, satellite DNA, segmental duplications, simple repeats, and VNTRs. A positive value indicates elevated performance within the annotated regions relative to the technology-wide average. A negative value indicates reduced performance. Annotations derived from RepeatMasker and UCSC tracks on GRCh38. B) Log fold change of recall (top) and precision (bottom) stratified by tandem-repeat subtype. STR loci (left) were binned by motif unit length (2, 3, 4, 5, and ≥6 bp). The 1 bp homopolymer class was absent from the reference catalog used. VNTR loci (right) were binned by reference-locus length (<200 bp, 200 to 500 bp, 500 bp to 1 kb, 1 to 5 kb, >5 kb). Each SV in the benchmark was assigned to its nearest STR or VNTR locus (within 100 bp) using the STR and VNTR catalogs from Lu et al. (2023)

**Fig. S6: Novel machine learning tool reliably distinguishes true variants from artifacts.** A) Precision-recall curves for *dicast* when only receiving the unfiltered callsets from the five SRS-based callers as input candidate variants and not an additional population catalog. B) Number of TP and FP variants across different SRS-based detection methods and size categories. *N* depicts the number of TP variants in the respective benchmark dataset. Considered are deletions, insertions, and duplications. C) Detection performance for deletions (N=101) and simple insertions (N=57) within challenging medically relevant genes (CMRG) as defined by Wagner et al., 2022.

**Fig. S7: Manually confirmed calls with high *dicast* prediction (0.998) and clear evidence in both short- and long-read sequencing.** Visualizations were created with cuban (See Fig. S14).

**Fig. S8: Manually confirmed call with medium *dicast* score (0.572) with conflicting evidence short- and long-read evidence.** Visualizations were created with cuban (See Fig. S14).

**Fig. S9: Manually confirmed call with low *dicast* score (0.1399) with inaccurate breakpoints.** Visualizations were created with cuban (See Fig. S14).

**Fig. S10: Visualization of the regulatory region affected by the NMD01-NMD03 deletions generated with the Lucid Genome Suite.** The figure shows several tracks of relevant coding and non-coding annotations starting with TAD annotations derived from our fibroblast Hi-C analysis and Enhancer signatures from ENCODE connected to the ENSEMBL gene they most likely regulate through the Hi-C interaction frequencies. The following tracks show the variants (SVs, SNVs and TRs) in all samples of the NMD cohort. The canonical transcript of the gene in focus (HNRNPA1) is shown in the next track. Finally, several additional regulatory tracks are included: CTCF binding sites, enhancer and promotor signatures derived through ChIP-seq experiments conducted on myotube samples (ENCODE), VISTA enhancers and TAD boundaries called with various resolutions.

**Fig. S11: Visualization of the regulatory region affected by the NMD04 deletion generated with the Lucid Genome Suite.**

**Fig. S12: Visualization of the regulatory region affected by the NMD05 deletion generated with the Lucid Genome Suite.**

**Fig. S13: Visualization of the regulatory region affected by the AF deletions generated with the Lucid Genome Suite.** The tracks include TADs derived from cardiomyocytes, enhancer signatures from the same cell type connected through Hi-C interaction frequencies to the genes they most likely regulate. In the last part of the figure all VISTA enhancers and those specifically active in heart cell-types in combination with TAD boundaries of various resolutions are shown. The remaining tracks are described in Fig S8.

**Fig. S14: Architecture of cuban plots.** Top tracks depict coverage, repetitive elements insert size outliers and discordant read pairs across the whole length of a given variant. The dotted red line within the coverage track indicates the chromosomal average coverage. Discordant read pairs include pairs in reverse-forward (dark blue), forward-forward (green) and reverse-reverse (orange) orientation. The bottom segment visualized CIGAR strings in two 200bp windows around each breakpoint. Light gray indicates a match/mismatch between the read and the reference, while green indicates soft-clipped bases. A black grid overlay indicates a split-read. Connections between reads around each breakpoint are shown between the two windows. Black lines indicate two segments of a split-read, while red lines connect two mates of the same read pair.

**Fig. S15: Sequence and functional context of manually confirmed SV candidates.** This figure summarizes the genomic context of the set of manually confirmed *dicast* benchmark deletions and duplications. A) Sequence context at SV breakpoints. For each confirmed SV, we quantified whether exactly one breakpoint or both breakpoints fall within key repetitive sequence context classes or in unique regions; bars report the percentage of confirmed SVs per class and overlap mode. B) Functional annotation context. The plot shows, for deletions and duplications separately, the number of confirmed SVs overlapping coding sequence, limb-specific regulatory elements, generic regulatory elements, Hi‑C–derived chromatin features (TAD boundaries and gene–enhancer interaction annotations), and DEG regulatory windows, providing an overview of the functional evidence supporting candidate selection.
